# Retraction Note: High replicability of newly discovered social-behavioural findings is achievable

**DOI:** 10.1038/s41562-024-01997-3

**Published:** 2024-09-24

**Authors:** John Protzko, Jon Krosnick, Leif Nelson, Brian A. Nosek, Jordan Axt, Matt Berent, Nicholas Buttrick, Matthew DeBell, Charles R. Ebersole, Sebastian Lundmark, Bo MacInnis, Michael O’Donnell, Hannah Perfecto, James E. Pustejovsky, Scott S. Roeder, Jan Walleczek, Jonathan W. Schooler

**Affiliations:** 1 Department of Psychological & Brain Sciences, University of California, Santa Barbara, Santa Barbara, CA USA; 2 Department of Psychological Science, Central Connecticut State University, New Britain, CT USA; 3 Institute for Research in the Social Sciences, Stanford University, Stanford, CA USA; 4 Haas School of Business, University of California, Berkeley, Berkeley, CA USA; 5 Center for Open Science, Charlottesville, VA USA; 6 Department of Psychology, University of Virginia, Charlottesville, VA USA; 7 Department of Psychology, McGill University, Montreal, Quebec Canada; 8 Matt Berent Consulting, Sharon, PA USA; 9 Department of Psychology, University of Wisconsin–Madison, Madison, WI USA; 10 SOM Institute, University of Gothenburg, Gothenburg, Sweden; 11 McDonough School of Business, Georgetown University, Washington, DC USA; 12 Olin School of Business, Washington University in St. Louis, St. Louis, MO USA; 13 Educational Psychology Department, University of Wisconsin–Madison, Madison, WI USA; 14 Darla Moore School of Business, University of South Carolina, Columbia, SC USA; 15 Phenoscience Laboratories, Berlin, Germany

**Keywords:** Human behaviour, Human behaviour, Psychology

Retraction to: *Nature Human Behaviour* (2023) 8:311-319 10.1038/s41562-023-01749-9

The Editors are retracting this article following concerns initially raised by Bak-Coleman and Devezer^1^.

The concerns relate to lack of transparency and misstatement of the hypotheses and predictions the reported meta-study was designed to test; lack of preregistration for measures and analyses supporting the titular claim (against statements asserting preregistration in the published article); selection of outcome measures and analyses with knowledge of the data; and incomplete reporting of data and analyses.

Post-publication peer review and editorial examination of materials made available by the authors upheld these concerns. As a result, the Editors no longer have confidence in the reliability of the findings and conclusions reported in this article. The authors have been invited to submit a new manuscript for peer review.

All authors agree to this retraction due to incorrect statements of preregistration for the meta-study as a whole but disagree with other concerns listed in this note.
